# A comprehensive study on cervical cancer diagnosis using deep learning and artificial intelligence techniques

**DOI:** 10.3389/frai.2026.1943994

**Published:** 2026-09-25

**Authors:** Nilasree Kannadoss, Kumar Rangasamy

**Affiliations:** School of Computer Science and Engineering, Vellore Institute of Technology, Chennai, Tamil Nadu, India

**Keywords:** artificial intelligence, cervical cancer, class imbalance, early diagnosis, federated learning, healthcare AI applications, machine learning, medical imaging

## Abstract

Cervical cancer is a significant health problem across the globe, especially in nations with low or middle incomes, where access to early screening is limited and contributes to high mortality rates. Traditional diagnostic methods, including Papanicolaou smears (Pap smears) and Human Papillomavirus testing (HPV testing), often suffer from accessibility constraints, lower accuracy, and scalability issues. Recent advancements in artificial intelligence (AI) and Machine Learning (ML) have exhibited significant efficacy by improving detection accuracy and streamlining the diagnostic procedure. This review discusses AI-driven methodologies, including deep learning models (EfficientNetB0, Cervical Net), hybrid frameworks, and ensemble techniques. This study examines important obstacles related to class imbalance, data privacy, and model interpretability while also presenting a solution known as the Adaptive Synthetic Minority Over-Sampling Technique combined with Tomek Links (Adaptive SMOTE Tomek) for data augmentation and federated learning (FL) for secure, distributed AI model training. The outcomes emphasize AI’s vital role in improving cervical cancer screening and correspond with the World Health Organization’s (WHO) 2030 goals for cervical cancer eradication. Future research should focus on real-world validation, improved generalization, and the integration of AI in clinical settings to ensure widespread usage and reliability. This work is useful for medical professionals in cervical cancer diagnosis, in addition to traditional screening methods.

## 1 Introduction

Cervical cancer, a treatable and preventable condition when detected early, is a threat worldwide as a result of infection with high-risk strains of human (HPV) Cibula et al. (2023). It ranks as one of the top four cancers among women globally, with the World Health Organization (WHO) documenting approximately 604,000 new cases and 342,000 fatalities in the year 2022. The management of cervical cancer is a significant concern in low- and middle-income countries (LMICs), accounting for 87% of all cervical cancer worldwide, Castle et al. (2021). They comprise poor health system infrastructure, insufficient screening activities, low health literacy, and cultural practices discouraging women from reporting to screening programs. Screening is crucial because cervical cancer progresses from pre-malignant dysplasia to invasive cancerous lesions over several years. HIV-positive women have a significantly higher incidence of cervical cancer D’Oria et al. (2022). There is a need for enhanced and more regular screening of HIV-positive women, which is difficult due to the lack of resources, particularly in low- and middle-income countries (LMIC) Obeagu and Obeagu (2023). However, insufficiently trained human resources, inconsistencies in quality assurance and delivery, and logistical challenges hinder their application in developing countries. Using artificial intelligence (AI)-based diagnostic apps as an example, new prospects for enhanced screen performance, accuracy, and coverage have been incorporated in contemporary medicine today, Mehmood et al. (2021). These technologies employ analysis of images and algorithmic learning to identify anomalies and reduce reliance on the human angle on such matters. However, there are some challenges when implementing such technologies, such as high costs, scarce availability of annotated datasets, or resistance to incorporating the computer systems in the list of clinical workflows Cheng et al. (2021). To address these challenges, we propose interventions such as awareness campaigns in public health, infrastructure development, and scaling up new solutions tailored to low- and middle-income countries (LMICs). Global commitment to intensifying cervical cancer prevention, screening, and treatment is necessary for promoting an equitable target for eliminating cervical cancer, which has been set by the World Health Organization (WHO) to be achieved by 2030 Desai et al. (2022).

Numerous challenges are associated with the early detection of cervical cancer, and their existence can result in difficulties during the process of the design and development of powerful diagnostic tools Wentzensen et al. (2021). It is significant to point out one fundamental limitation in medical databases: class distributions are highly imbalanced, where benign cases greatly outnumber malignant cases. This is a situation likely to result in the biasing of the machine learning models towards the majority class, thus having a significant impact on sensitivity and specificity in classifying minority cases Kanavati et al. (2022). Consequently, malignant cases are likely to be misclassified, thus putting patients at risk of poor prognosis through delayed diagnosis. In addition, the issue of data privacy is a problem, as health-related data involves individual information that is protected by law, such as the Health Insurance Portability and Accountability Act (HIPAA) and the General Data Protection Regulation (GDPR). The laws complicate decentralization in medical data, and this presents numerous challenges in accessing and exchanging the large-scale data required in the effective training of machine learning Ma et al. (2022). In addition, architectures in decentralized data settings present challenges in standardization, thus introducing variability that can be detrimental to the performance of models. Further, staging of cervical cancer is highly challenging due to the faint difference between cancerous and precancerous cytologic changes in the cervix Chen et al. (2023). Conventional supervised machine learning models have poor ability in transferring learning between different datasets due to their high dimensionality in general as well as variability in medical images Mohammadi et al. (2021). This is further compounded by the lack of data with sufficient annotation due to the time-consuming process of annotating medical images and the need to engage healthcare workers Kurita et al. (2023). The second challenge is the model’s interpretability, an important requirement in medicine where the underlying reason for the decision should be explained and understandable to doctors and other health workers Salehi et al. (2023). Solving such complex problems requires special techniques to facilitate management of biased data, protect the privacy of users, and improve the performance of classification models Zhang et al. (2023).

The article presents the state-of-the-art advancements in detecting and classifying cervical cancer, with an enabling role served by artificial intelligence (AI) and Machine Learning (ML). Such technologies assume prime importance in improving the accuracy of diagnosis and screening efficiency, a prime cause of cancer mortality among women. Traditional techniques such as Papanicolaou smears (Pap smears), Human Papillomavirus (HPV) testing, and Visual Inspection with Acetic Acid (VIA) have been effective but have lower sensitivity, specificity, and scale. AI and ML, contrastingly, hold revolutionary promise: the ability to use large-scale datasets to train models that could identify abnormalities with high accuracy. Advanced AI models such as Convolutional Neural Networks (CNNs), transformers, and hybrid models are superior in feature extraction and multi-class classification and can efficiently process imbalanced datasets. In addition, ML algorithms such as random forests, Support Vector Machines (SVMs), and ensemble methods also contribute to early detection by analyzing the risk factors and cytological patterns. Such technologies reduce the need for specialized people in their deployment. The research study also enlists challenges such as data privacy, unavailability of annotated data, and seamless integration into clinical workflows. It mentions upcoming directions on federated learning (FL) as a solution to achieve private model training and adaptive sampling techniques to handle class imbalances. The article enlightens such advancements in favour of AI/ML for attaining the World Health Organization’s (WHO’s) goal of eliminating cervical cancer as a public health problem by 2030. Figure 1 describes the different types of screening methods, their symptoms, and advanced methods of cervical cancer screening.

**Figure 1:**
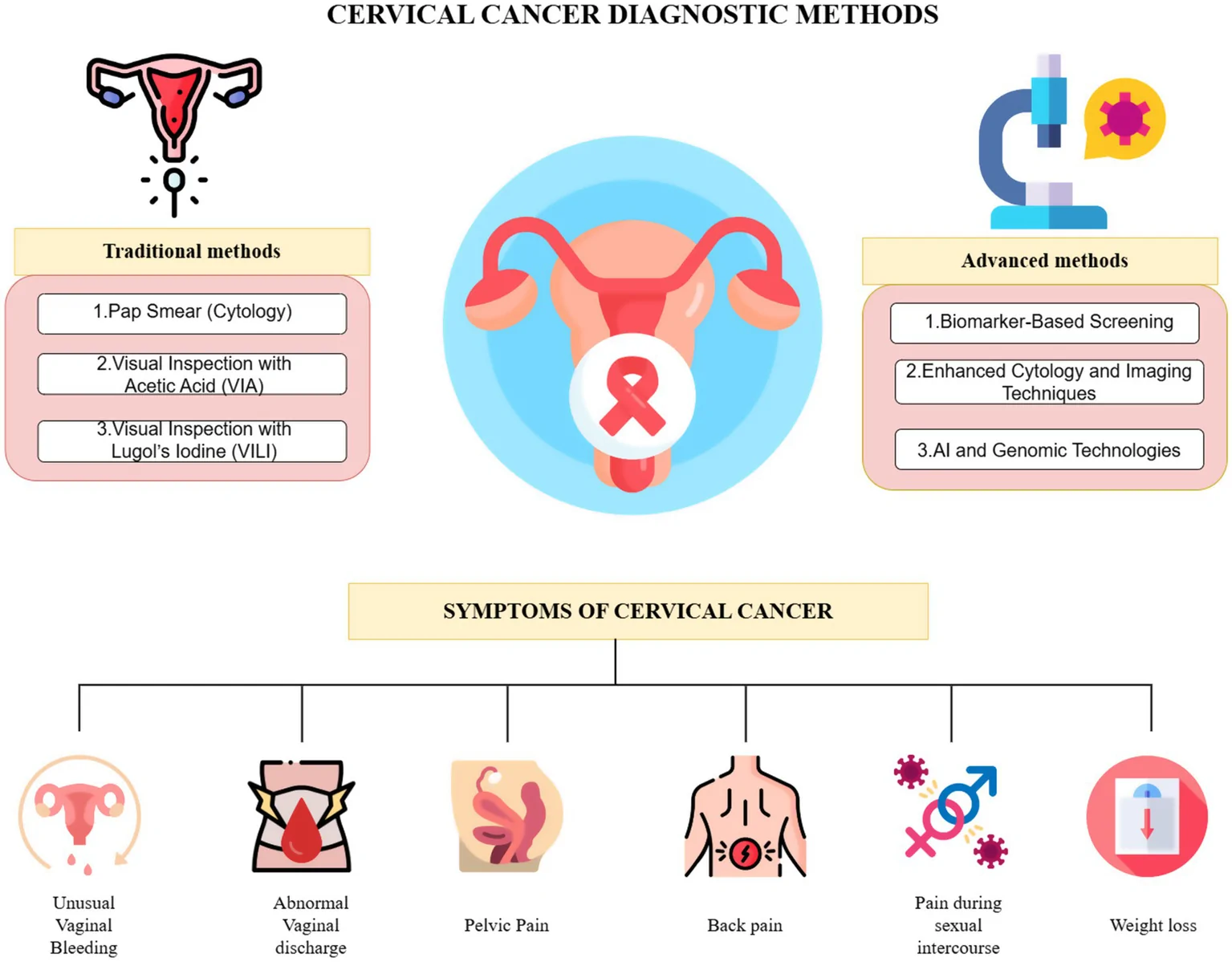
Cervical cancer diagnostic methods and its symptoms.

### 1.1 Research objectives

- To evaluate the effectiveness of artificial intelligence (AI) and Machine Learning (ML) techniques in improving the results and performance of cervical cancer detection and classification than traditional diagnostic methods.
- To identify the key challenges in implementing artificial intelligence (AI) and Machine Learning (ML) technologies in cervical cancer screening, particularly in low- and middle-income countries (LMICs).
- To explore the potential of AI-driven approaches in overcoming issues related to data privacy, class imbalance, and model interpretability in cervical cancer detection.

### 1.2 Research questions

- How do artificial intelligence (AI) and Machine Learning (ML)-based diagnostic tools compare to traditional methods (such as Papanicolaou smears (Pap smears) and Human Papillomavirus (HPV) testing) in terms of accuracy, sensitivity, and specificity for detecting cervical cancer?
- What are the primary barriers to the adoption and successful integration of artificial intelligence (AI)-based diagnostic tools in cervical cancer screening programs in Low- and Middle-Income Countries (LMICs)?
- How can artificial intelligence (AI) and Machine Learning (ML) techniques address the challenges of class imbalance, data privacy concerns, and model interpretability in cervical cancer detection, and what impact do these solutions have on model performance in real-world clinical settings?

## 2 Research methodology

The research uses a Systematic Literature Review (SLR) to carefully look at the latest methods for detecting and classifying cervical cancer, focusing on how Machine Learning (ML) and Deep Learning (DL) can help, while also considering privacy through federated learning and addressing data imbalance using the Adaptive Synthetic Minority Over-Sampling Technique (SMOTE) Tomek. We methodically selected peer-reviewed studies from the Scopus, Springer, IEEE Xplore, and Elsevier databases between 2021 and 2024. We employed keywords such as “Cervical Cancer Detection,” “Deep Learning,” and “Machine Learning” to guide the search, resulting in the retrieval of a focused dataset of 63 studies. The review process followed the Preferred Reporting Items for Systematic Reviews and Meta-Analyses (PRISMA) guidelines to make sure everything was clear, starting from 920 records and narrowing down to relevant and high-quality peer-reviewed studies. Assessments were provided for methodology, performance measures like accuracy, sensitivity, and specificity, and scalability. We prioritized the selected literature to ensure its focus on enhancing early cervical cancer detection and classification. This review is in line with the latest PRISMA 2020 guidelines of systematic review and meta-analysis reporting. It examines ML DL hybrid diagnostic systems for identifying cervical cancer, highlighting privacy preserving (federated learning) and imbalance handling techniques (Adaptive SMOTE-Tomek).

### 2.1 Selection criteria and information sources

They systematically searched four leading academic sources: IEEE Xplore Scopus SpringerLink, and ScienceDirect (Elsevier). It was from January 15 2024 to February 10, 2024 that they carried out their search for references related to publications between January 1, 2021, and December 30, 2024.

For full algorithmic reproducibility, search phrases were separately crafted for each database using Boolean operators (AND, OR), truncation wildcards (*), and field-targeted tags. The main conceptual elements brought together:

1. *Disease Domain*: (“cervical cancer” OR “cervical lesion*” OR “pap smear” OR “cervicography” OR “colposcopy”)
2. *Computational Approach*: (“deep learning” OR “machine learning” OR “artificial intelligence” OR “convolutional neural network” OR “vision transformer” OR “federated learning”)
3. *Target Optimization*: (“class imbalance” OR “SMOTE” OR “data privacy” OR “segmentation” OR “classification”)

#### 2.1.1 Explicit search syntax by database

- *IEEE Xplore*: ((“Document Title”: “cervical cancer” OR “Abstract”: “cervical cancer”) AND (“Abstract”: “deep learning” OR “machine learning” OR “federated learning”) AND (“Abstract”: “classification” OR “imbalance” OR “privacy”))
- *Scopus*: TITLE-ABS-KEY((“cervical cancer” OR “pap smear” OR “colposcopy”) AND (“deep learning” OR “machine learning” OR “federated learning”) AND (“class imbalance” OR “SMOTE” OR “classification”)) AND PUBYEAR > 2020
- *SpringerLink*: (“cervical cancer” OR “pap smear”) AND (“deep learning” OR “machine learning”) AND (“class imbalance” OR “privacy”) within Computer Science and Medicine collections (2021–2024).
- *ScienceDirect*: Title-Abs-Key: (“cervical cancer” AND (“deep learning” OR “machine learning”) AND (“classification” OR “imbalance”)) filtered by Journal Articles/Conference Papers (2021–2024).

### 2.2 Data sources

Peer-reviewed conference proceedings and journal articles focusing on various machine learning (ML) and deep learning (DL) methods related to cervical cancer detection and classification were collected from highly ranked academic databases, including Scopus, Springer, IEEE Xplore, and Elsevier (Gururaj et al., 2025). Benchmark data, such as the Herlev and Papanicolaou (Pap) smear, are assured for practical usage; these data are real instances related to cervical cancer classification. Such data have been crucial for ensuring the efficiency of the models and algorithms in question. Industry literature from respected agencies, such as the World Health Organization (WHO), along with specialized healthcare artificial intelligence (AI) and medical imaging-focused journals, has also been part of the knowledge base. A detailed search has been conducted using specific terms like “Cervical Cancer Machine Learning (ML) Models,” “Deep Learning,” and “Hybrid Deep Learning (DL) cervical cancer,” to ensure that important topics such as privacy, performance improvement, and class imbalance in cervical cancer classification models are well covered.

### 2.3 Inclusion and exclusion criteria

The inclusion criteria focused on the selection of very high-quality, peer-reviewed journal articles and conference proceedings from credible databases like Scopus, Elsevier, Springer, and IEEE Xplore. The studies included were from 2021 to 2024, written in English, and allowed full-text access. We specifically focused on class imbalance, cervical cancer detection, and classification, giving preference to machine learning (ML), deep learning (DL), and hybrids. Exclusion criteria eliminated non-peer-reviewed papers, grey literature, non-English articles, inaccessible full texts, and publications not focusing on cervical cancer detection using machine learning (ML) or deep learning (DL). The studies that fell outside the window of publication dates between 2021 and 2024 or were not relevant to the objectives of the study were excluded to ensure a focused, relevant dataset for analysis. Figure 2 represents the inclusion and exclusion criteria.

**Figure 2:**
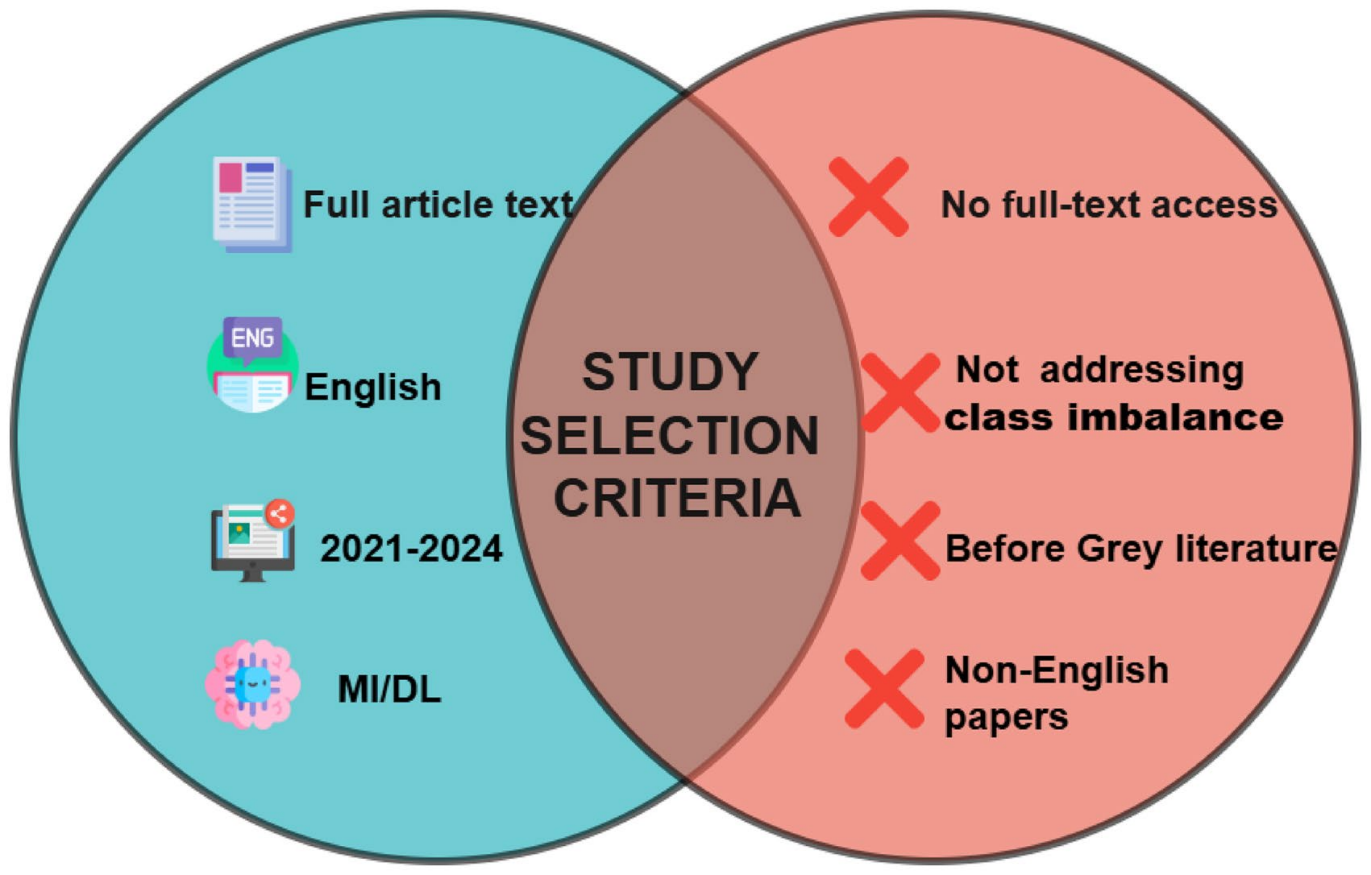
Inclusion and exclusion criteria.

### 2.4 Systematic review process

Our systematic review process was conducted with methodological rigor and complete disclosure, in line with the Preferred Reporting Items for Systematic Reviews and Meta-Analyses (PRISMA) guidelines. The search in the database yielded 920 records, we took out 210 duplicates. Next, we examined 710 full texts on their titles and abstract level. After the first step, about 370 papers were rejected as they were not directly related to machine learning and deep learning applications for detecting and classifying cervical cancer.

In the next phase, we checked the content of all documents thoroughly to find out if they fit the scope of our project and if they were scientifically, and clinically accurate. The reasons why 277 studies have been removed can be listed as; 142 used neither machine learning nor deep learning techniques, 85 papers did not tackle class imbalance and/or private architectures, the remaining 50 provided either no detailed validation or performance indicators. At the end of the process 63 well-written, high-quality articles have been selected in line with all the required criteria. They were the basis for a series of studies including: ML\DL workflow improvements, privacy-preserving structures, and class imbalance strategies (see Figure 3).

**Figure 3:**
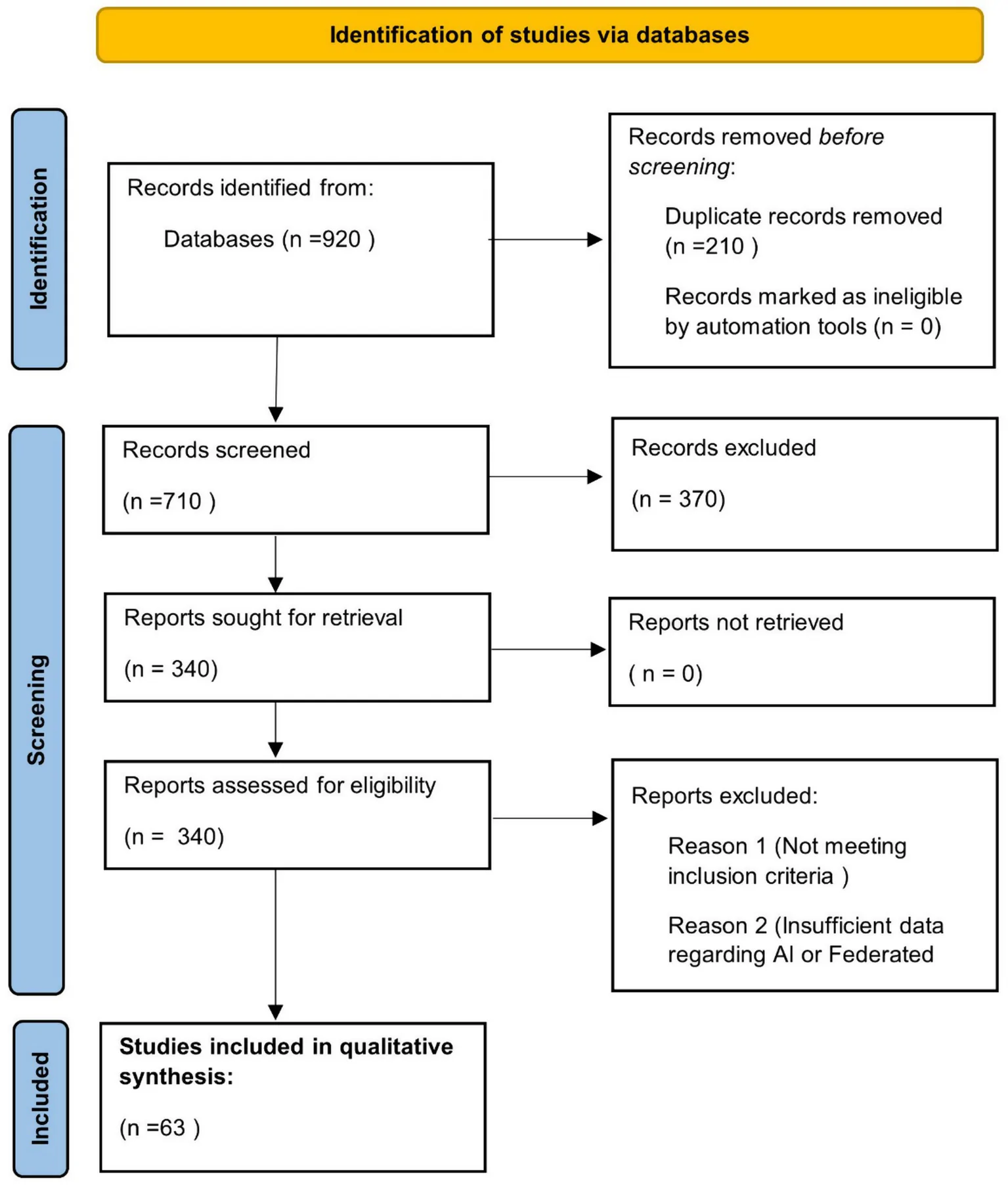
PRISMA framework.

### 2.5 Search results

Systematic search through the initial *920 records* reveals *63 articles*. The purpose of these papers is to show various applications in ML and DL for detection and classification tasks that were focused on improvements in methodology addressing class imbalance, data privacy, and high predictive accuracy. Repeated was the application of the privacy-preserving frameworks, with their ability to perform collaborative training of models based on distributed datasets in a privacy-compliant fashion. Many papers discussed novel approaches like transfer learning and ensemble techniques, which improved the generalization of the model and performance across various datasets. Hybrid approaches that combined ML and DL methods became effective solutions to leverage the strengths of multiple algorithms for better robustness and accuracy in predictions. The review showed how these advances collectively contribute to improving early detection and classification accuracy, highlighting the critical role of ML and DL in addressing challenges associated with imbalanced and distributed healthcare data.

### 2.6 Methodological quality and critical appraisal framework

To check the technical validity and possible risk of bias in all the included studies (*N* = 63), a structured 5-domain critical appraisal checklist for medical AI and digital diagnostic systems was used by two independent researchers to appraise each paper along five important critical analytical areas and the levels of concerns were rated as low, some, or high/unclear:

Domain 1 (D1) Evidence/Data Source: This domain evaluates the reliability of the dataset and the suitability of the sample size. One key factor is that ground-truth data used to train the models (like Pap smear images, whole-slide images, or CT scans) should also be clinically or pathologically verified. Finally, the demographic distribution of the dataset should be transparently described.

Domain 2 (D2) Methodological Transparency: This domain checks to see if one can reproduce the algorithm by simply using the code available. The network architecture should be clearly described, with hyperparameters, loss functions, and pre-processing steps.

Domain 3 (D3) Validation/Evidence Appraisal: This domain evaluates the validation protocol: whether a training set, a validation set, and a testing set can be clearly distinguished or whether a testing set is independent of the training/validation set. It should also be mentioned whether cross-validation is used, and if so, whether it is a good design. Finally, it is checked and reported that there is no leakage in the data when data augmentation is performed, or sampling is split.

Domain 4 (D4) Evaluation/Outcome Assessment: This domain checks whether all the metrics that are typically used in evaluating a diagnostic algorithm are included in the work or if the selection of some metrics might have been made simply because they were reported earlier in the field and not because they best served the purpose. For example, metrics like Accuracy Sensitivity Specificity, ROC-AUC, F1-score, and Dice Similarity Coefficient have to be reported in a complete manner to assess the effectiveness of the method.

Domain 5 (D5) Reporting/Limitations / Applicability: This domain evaluates how clearly and comprehensively the authors acknowledge the shortcomings of their study, the clinical context or setting that inspired their work or in which their system is expected to be applied, and the practical deployment constraints of their system like hardware limitations, computational demands, and user interaction.

Out of the 63 studies reviewed, 35 (55.6%) were overall low concern papers, as they were accompanied by very good validation procedures and open descriptions of their pipeline. The other 28 papers (44.4%) either raised some concerns or lacked clarity in certain areas, usually because operational limitations had not been disclosed in Domain 5 or the external validation was not reported sufficiently in Domain 3. Table 1 contains the critical appraisal summary across reviewed papers.

**Table 1:** Methodological critical appraisal summary across reviewed papers (*N* = 63).

Assessment domain	Low concern [*n*, (%)]	Some concern [*n*, (%)]	High concern/unclear [*n*, (%)]
D1: Evidence/Data Source	44 (69.8%)	17 (27.0%)	2 (3.2%)
D2: Methodological Transparency	49 (77.8%)	12 (19.0%)	2 (3.2%)
D3: Validation/Evidence Appraisal	16 (25.4%)	41 (65.1%)	6 (9.5%)
D4: Evaluation/Outcome Assessment	55 (87.3%)	6 (9.5%)	2 (3.2%)
D5: Reporting/Limitations/Applicability	12 (19.0%)	48 (76.2%)	3 (4.8%)
Overall methodological risk rating	35 (55.6%)	26 (41.3%)	2 (3.2%)

## 3 Literature review

### 3.1 Advancements in automated cervical cancer screening using DL and ML

Cervical cancer is still among the principal killer diseases in women, especially in developing nations where there is a dearth of adequate equipment for screening. To overcome these challenges, some authors have suggested the following proposed automated systems: deep learning (DL) and machine learning (ML) techniques for cervical cancer detection. A study by Habtemariam et al. (2022), who used MobileNetv2 and You Only Look Once (YOLOv3), achieved excellent mean average precision in identifying the region of interest (ROI) from 4,005 colposcopy and 915 histopathology images. Cervix type classification and cervical cancer classification on EfficientNetB0 models showed excellent accuracies, respectively, which can supplement early diagnosis on a limited budget. Diniz et al. (2021) focused on studying Pap smear images and used a step-by-step classification method with the Random Forest approach, which improved the detection of cell problems and reduced the workload for cytopathologists. Implemented and tested on the Herlev and CRIC databases, the system enhanced initial recognition and helped cytopathologists diagnose cervical lesions more effectively. Later studies by Park et al. (2021) looked at how accurately cervical cancer can be identified using machine learning (ML) and deep learning (DL) models on 4,119 cervicography images, automatically identifying ML and DL models like extreme gradient boosting (XGB), support vector machines (SVM), and ResNet-50. It was noted that the Deep Learning (DL) model has a higher Area Under the Curve (AUC) of 0.97 than the Machine Learning (ML) model, proving that Deep Learning results in higher accuracy in cervical cancer identification (Gururaj et al., 2025). In the same year, Alquran et al. (2022a) introduced a Computer-Aided Diagnosis (CAD) system that used images from Pap smears, utilizing the Cervical Net Deep Learning (DL) structure with ShuffleNet features, which achieved 98.1% accuracy compared to the Support Vector Machine (SVM) classifier, showing that deep learning can improve cervical cancer screening. Additionally, Youneszade et al. (2023) demonstrated that deep learning (DL) can eliminate human mistakes in automated cytology and colposcopy images during cervical cancer screening, improving the accuracy of detecting different stages of cervical cancer.

These studies point to the revolutionary aspect of machine learning (ML) and deep learning (DL) in cervical cancer screening. These techniques, which range from Pap smear examination to cervicography, significantly improve the efficiency and accuracy of cervical cancer detection. By reducing mistakes made by people and using deep learning (DL) and machine learning (ML) to analyze images automatically, we can help health workers manage their tasks better, allowing for quicker detection of cervical cancer in places with fewer resources. Utilizing more complicated models like MobileNetv2, EfficientNetB0, ResNet-50, and Cervical Net has excellent prospects of going towards the improvement of early-stage cancer detection and further improving the treatment outcome for the patient. Figure 4 represents the Cervical Cancer Detection Studies.

**Figure 4:**
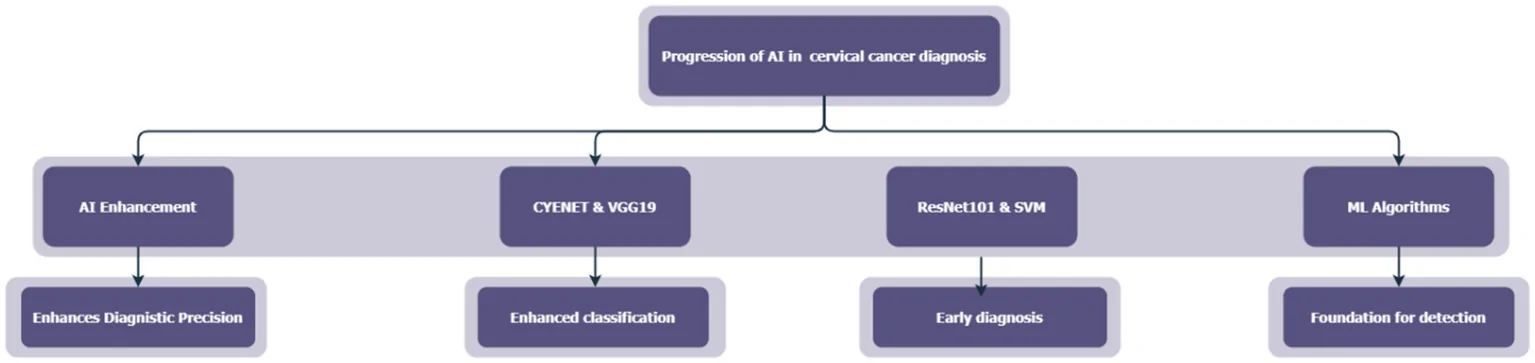
Cervical cancer detection studies.

Table 2 shows the advancements in cervical cancer detection using artificial intelligence (AI) and Deep Learning (DL) models for greater accuracy and efficiency. Techniques such as MobileNetv2-YOLOv3, EfficientNetB0, and Cervical Net achieved high accuracy, particularly in low-resource settings. DL-based Computer-Aided Diagnosis (CAD) systems mechanized segmentation and classification processes with automation, reducing workloads and effectively improving lesion detection and screening efficiency across a variety of imaging datasets. Recent advances have highlighted the growing role of artificial intelligence, machine learning, and deep learning as intelligent tools for supporting healthcare decision-making and improving automated healthcare systems (Jain et al., 2025).

**Table 2:** Comparison of ML and DL approaches for cervical cancer detection.

Study	Method	Dataset	Results	Key findings
Habtemariam et al. (2022)	MobileNetv2-YOLOv3 + EfficientNetB0	4,005 colposcopy, 915 histopathology images	Good accuracies	Developed a robust, automated system for cervical cancer classification, effective in low-resource settings
Diniz et al. (2021)	Random Forest classifier + hierarchical classification	Herlev and CRIC databases (Pap smear)	High accuracy for cell lesion detection	Reduced cytopathologist workload, improved lesion screening efficiency
Park et al. (2021)	ResNet-50	4,119 cervicography images	AUC = 0.97, outperforming ML models	DL models, particularly ResNet-50, outperformed ML models, offering superior accuracy for cervical cancer detection
Alquran et al. (2022a)	Cervical Net + ShuffleNet features + SVM	Pap smear images	98.1% accuracy	High accuracy in cervical cancer detection using integrated DL models
Youneszade et al. (2023)	DL-based CAD systems	Cytology and colposcopy images	Enhanced classification accuracy	DL-based CAD systems improve cervical cancer screening by automating image segmentation and classification

### 3.2 AI and DL techniques for cervical cancer screening

Cervical cancer is still a major concern in the world today, and therefore early screening is essential if one is to come out of the disease. New developments in artificial intelligence (AI) and Machine Learning (ML) over the years have presented innovative methods for increasing the success rates of cervical cancer diagnosis, shortening the time for diagnosis, and relieving pressures on carrying out the diagnosis from the health facilities. Several studies provide an insight into the changing dynamics of the use of AI and ML in the context of problems like scarcity of capital, ambiguity in interpretation, and the requirement of skilled personnel. For instance, Hou et al. (2022) acknowledge that AI brings proposals for reduction of subjective bias, reliance on acquired professionals to be reduced, and hence enhanced precision in diagnosis due to automation of the analysis. In colposcopy image analysis, Chandran et al. (2021) introduced CYENET, which was much more efficient than traditional diagnostic methods, achieving an accuracy of 92.3%, sensitivity of 92.4%, and specificity of 96.2%. Examples listed above show that the ensemble model is useful in the management of cervical cancer screening. Similarly, Pacal and Kılıcarslan (2023) conducted a study comparing deep learning (DL) models and found that Vision Transformer (ViT) models worked a bit better than convolutional neural networks (CNNs) for automating cervical cancer diagnosis. This means there is a need to investigate other architectures, like ViTs, for enhancing diagnostic performance and thereby reducing mortality.

Apart from the application of image-based analysis, various studies have explored the potential of applying machine learning (ML) algorithms to create models for cervical cancer screening based on patient features. Jahan et al. (2021) applied 8 different ML algorithms and concluded that Multilayer Perceptron (MLP) is the most precise for predictive analytics. Nevertheless, Alquran et al. (2022b) presented a new ResNet101 and Support Vector Machine (SVM)-based hybrid system for classifying Pap smear images. This system has an overall training accuracy efficiency of 97.3 percent, demonstrating that in the context of automated cervical abnormality detection, diagnostic errors can be reduced to a minimum.

All these studies collectively outline the revolutionary role of AI and ML in the diagnosis of cervical cancer. These technologies promise to revolutionize cervical cancer detection and early treatment by automating image classification, reducing issues like variability in data, and enhancing traditional screening practices. The integration of AI-driven systems in clinical practice has enormous benefits, such as the enhancement of accuracy, workflow optimization, and minimizing the workload of medical professionals. However, while these developments promise a lot, challenges remain. Some of the areas that require enhancement include data quality, model generalizability, ethical issues, and the capacity to integrate AI seamlessly into clinical workflows to maximize impact in real-world settings. Additional research is also required to standardize AI systems, facilitate interoperability between different healthcare environments, and protect patient privacy. With these challenges addressed, there is a likelihood that AI and ML will be applied on a large scale in screening and diagnosing cervical cancer, thereby decreasing the worldwide burden of the disease and saving thousands of lives. Some methods work better than traditional ones by being very accurate, like CYENET, which is based on ViT, and ResNet101 with SVM classifiers. MLP algorithms optimize the prediction of early risk. AI further reduces dependency on specialized personnel, raising the level of the diagnostic workflow. Figure 5 represents the AI and DL taxanomy for cervical cancer.

**Figure 5:**
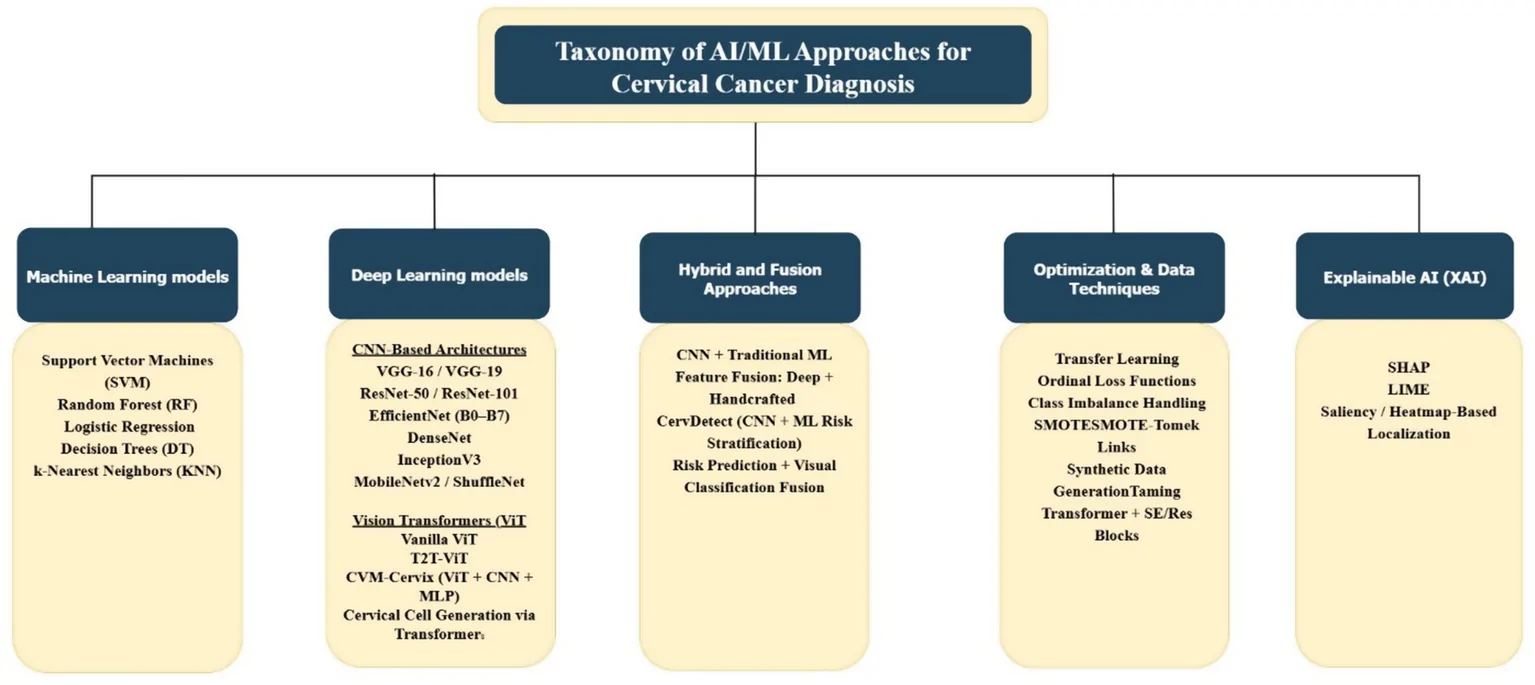
AI and DL taxonomy for cervical cancer.

### 3.3 Hybrid and ensemble DL approaches for cervical cancer classification

The cervical cancer diagnosis has been significantly improved with the advent of DL and hybrid methods, bringing about higher accuracy, efficiency, and effectiveness of the diagnostic processes. The major deficits of conventional methods are inter-observer variation, higher cost compared to automated systems, and duplication of automated techniques, which calls for wider use. Combining these computing methods using hybrid and deep learning techniques has been shown to solve these problems, making it possible to create a better cervical cancer screening system. Another such contribution in the same direction is the method adopted by Mohammed et al. (2022), wherein CNN features from VGG-16 and GoogLeNet are combined with features. The fusion method achieved perfect accuracy, showing how artificial intelligence can help improve diagnosis accuracy and address the lack of cytologists, as well as provide low-cost diagnostic tools, especially in developing countries. Similarly, Waly et al. (2022) created the IDCNN-CDC model, which uses advanced steps to prepare data, like Gaussian filtering and segmentation with the Threshold Entropy Differential Flower Optimization (TE-DFO) algorithm. SqueezeNet features are classified using a weighted ELM, and good results are obtained when a multi-stage approach is used for diagnosing cervical cancer with the Herlev Pap smear database.

With this, Rahaman et al. (2021) offered hybrid feature fusion methods that can potentially bypass the barrier of manual screening. DeepCervix employs a hybrid method of deep feature fusion to make classifications dependent and address the issue of unevenness in data. Ensemble learning methods were improved by Pramanik et al. (2022), with emphasis on a fuzzy distance-based ensemble method that leveraged the power of three state-of-the-art models. By combining predictions and simplifying the results, the model reached an accuracy of 96.96%, which is better than using single classifiers, showing that ensemble methods can provide better diagnostic results. At the same time, Alsubai et al. (2023) presented a lightweight and efficient CNN architecture for cervical cell classification with the SIPaKMeD dataset. The architecture achieves an accuracy of 91.13% in telling apart precancerous abnormalities from healthy cells, making it a lightweight and easy-to-use option for detecting cervical cancer.

The studies collectively illustrate how hybrid approaches and deep learning architectures overcome the serious limitations in cervical cancer detection. The approaches integrate hand-designed features with deep feature representations and ensemble methods to improve diagnostic accuracy, reliability, and effectiveness. In addition, they overcome issues in class imbalance, limited availability of expert cytologists, and image quality variability. In the long run, these advances have the potential to transform the landscape of clinical applications by enabling the deployment of scalable, automated, and thus robust diagnostic tools that facilitate early detection for cervical cancer management, ultimately benefiting patient outcomes and reducing health disparities.

### 3.4 Advanced techniques and ML/hybrid approaches for cervical cancer detection

Emerging artificial intelligence and deep learning breakthroughs have brought profound transformations in cervical cancer detection and classification methods. They have become a very efficient way of offering new and more precise alternatives to traditional screening and diagnostic procedures. These technologies have also helped shorten the processing time, achieve greater accuracy, and improve accessibility, mostly in places where the healthcare system is unable to cope with a growing demand. Liu et al. (2022) brought a big advancement to cervical cancer classification techniques by proposing the CVM-Cervix structure. This paper describes a novel method of combining CNNs for local feature extraction with visual transformers for global feature extraction and taking both together with the advantages of each other. Feature fusion is then achieved by a multilayer perceptron. The model remains small yet highly accurate, which makes it appropriate for use in a healthcare centre setting. The role of post-processing is so significant that one can argue the whole process is almost effortless. As a result, it is easy to say that such techniques are more than just practical but also effective solutions when it comes to applications in real-life situations. On a related note, Mehmood et al. (2021) presented the CervDetect approach.

Albuquerque et al. (2021) described a nonparametric ordinal loss function that took into account the ordinal relationships between images. Based on experiments carried out on a clinical image set of cervical lesions, the novel function led to an 81.3 accuracy level in four-class classification of cervical cancer risk prediction, so emphasizing the importance of developing customized loss functions that are able to express the medical data complexities. Dhawan et al. (2021) stated that Deep Transfer Learning (TL) techniques have made it possible to overcome the challenge of scarce labeled data and the computational cost. Transfer learning on the top pre-trained models like InceptionV3, ResNet50, and VGG19 for the task of cervix-type classification achieved an accuracy of 96.1%. That high of accuracy is a step forward towards an efficient and accurate diagnosis with proper treatment regime formulation. Accurate identification and assessment of cervix type is key to determining adequate and timely medical management plans. Artificial intelligence has made cervical cancer screening Much more accurate in low- and middle-income countries, where traditional cytological methods are limited due to lack of skilled cytologists and availability of advanced diagnostic tools. Wu et al. (2024) emphasized the value of artificial intelligence (AI) in facilitating the interpretation of medical images depicting atypical cytology and CIN as a solution to both infrastructural and implementation barriers. These breakthroughs are essential for providing equitable access to cervical cancer screening and early interventions.

In addition to sophisticated deep learning methods, several papers have explored machine learning techniques as well as the use of hybrid models for the detection of cervical cancers. In fact, one of the breakthroughs is the Boruta-support vector machine model presented by Lilhore et al. (2022); here, Boruta analysis served the purpose of choosing the best cervical cancer features. Besides, the model made use of feature selection methods like Boruta as feature selection which was effective in reducing the dimensionality of the feature matrix and avoiding the threat of model over-fitting and it allowed only meaningful variables to influence the classification decision. In a different research, Alpan (2021) tested eight different machine learning methods and found that support vector machines performed the best with a 90.66% accuracy which indicates their strength in discrimination for the two categories of subjects. This finding demonstrates the strength of support vector machines in dealing with tough data and its possible application in improvement of cervical cancer diagnostics, mainly in regions where there is a lack of skilled cytologists.

Kruczkowski et al. (2022) proposed an optoelectronic sensor based classification system using the machine learning method to quickly classify tissues per its refractive index. Classifier based on sensor fusion, such as Random Forest, Extreme Gradient Boosting or convolutional neural network based classifier is fast and accurate in tissue classification and That means can be used to enable early staging of cervical cancer, assist in the reduction of invasiveness of such procedures and decrease the usage of expensive procedures through non-invasive sensor based system inclusion. The target is the realistic strategy in resource limited setting. Demonstrated improvements also show that machine learning is capable of accelerating diagnosis and, Because of this, increases the dependability of cervical cancer detection versus alternative techniques which may involve human error and bias. On top of that, recent advances in cervical cancer image processing and segmentation bring further improvements to the technique. For example, Al Mudawi and Alazeb (2022) tested several classifiers in combination with machine learning and found that random forests, decision trees and gradient boosting demonstrated a very high within-subject accuracy of 99%, with accounting for the space complexity. This highlights the wide possibility of optimization that can be achieved in real-world environment. Returned to such participants allowed their feedback incorporated into the research to be combined with the predictive model and so emphasize the need for participatory design in machine learning based healthcare application. In the same way, Desiani et al. (2021) focused their work on enhancement of Pap smear images, with aid of convolutional neural network used for segmentation with the use of k-nearest neighbors classification as well as artificial neural network as classification tools. Demonstrated sensitivity was over 0.854, specificity was over 0.874 and F1 score was over 0.595 in detecting abnormal cervical cells was demonstrated and the model was shown to be very reliable. Advanced image enhancement technology is used to improve image quality, so that abnormal and precancerous cells can be easily identified. This effectively raises the diagnostic accuracy.

These studies in total have proved the value of deep-learning and hybrid learning techniques, as well as image-processing algorithms, for the early identification of cervical cancer. The implementation of state-of-the-art neural network designs, pre-trained networks, ordinal loss functions, feature selection, sensor-driven solutions, and segmentation have demonstrated that such techniques can A lot enhance the quality, speed, and affordability of diagnosis, overcoming limitations of data scarcity and imbalance of classes, shortage of expert cytologists. By decreasing the scope of human interventions and raising the level of the model, these technologies stand out as the main factors that would allow us to achieve a very simple, cost-effective, easily-available, and trustworthy cervical cancer screening system besides the timely treatment, management of the patient, respectively. And, the development of innovative systems of this nature not only demands cross-disciplinary collaboration but also combines the expertise from machine learning, medical image processing, and the day- to-day practice of clinicians.

### 3.5 Advancements in cervical cancer screening: explainable AI, hybrid models, and transformer-based techniques

Recent advancements in the fields of machine learning and image processing have revolutionized the process of cervical cancer detection and classification significantly and also boosted precision, efficiency, and the capability to handle bulk data. A case in point among many is that in Pirovano et al. (2021), a study offered an explainable region classifier with a specific motive to tackle enormous pap smear images (as big as 100,000 × 100,000 pixels). The study achieved a high accuracy of 95.2% in classifying normal and abnormal cells, and achieved a Kappa score of 0.870, indicating perfect agreement between the predicted and actual classification. The method focuses on weakly supervised localization, allowing it to find abnormal cells linked to cancer with 80.4% accuracy, showing that machine learning models can not only classify images but also give understandable results for future clinical analysis. The technique is an additional step ahead for cervical cancer automation because it sidesteps large, complex dataset challenges and concurrently maintains accuracy while making sense out of the procedure. Srinivasan et al. (2023) explain a method that uses adaptive histogram equalization with cervical images and finite ridge let transform. Adaptive histogram equalization is helpful in enhancing the difference in image contrast, making the details, which play a crucial role in diagnosis, clearer. While in the case of the ridgelet transform, multi-scale information enhances the representation of image features crucial to detecting the cervical abnormalities’ irregularities. Superposition of these two image processing methods provides an even more powerful tool to detect cervical cancer and again eliminates minor faults that are typical in normal cervical exams.

Singh and Goyal (2023) introduce additional modifications, highlighting the application of a hybrid of segmentation and feature selection. With the application of extra tree classifiers to analyze benchmark datasets, the method had excellent accuracy when coupled with logistic regression and L1 regularization, although computational expense remains a problem. Hybrid methods that combine more than a single machine learning approach are turning out to be extremely effective since they tap into the strengths of multiple algorithms as well as improve the overall performance. However, we must overcome the computational expense of these models, particularly when scaling them for real-life clinical application. Notwithstanding these shortcomings, these models have a lot of potential in being extremely accurate and efficient cervical cancer screening tools, which would reduce workload for healthcare professionals as well as speed up diagnostic pipelines. Other developments have been in the area of overcoming restrictions on datasets, which has been a bottleneck to machine learning model performance over time. Zhao et al. (2022) tackle this issue by using a cervical cell generation model (Cervical Cell Generation-taming transformers) that employs advanced techniques like SE-blocks, MultiRes-blocks, and SMOTE-Tomek Links to balance datasets and enhance classification accuracy. The model showed a high accuracy on Herlev’s seven-class classification task. The dataset is balanced in a manner that the model is able to effectively reduce the risks of overfitting and even improve generalization, thus performing well on different public datasets. This procedure is especially useful in ensuring that machine learning models are trained using different datasets without bias towards any of the classes such that the trained models perform better in withstanding other clinical settings. Yu et al. (2021) proposed several models for classifying abnormal cervical cells in deep learning, and the fourth model included convolutional neural networks with inception modules and spatial pyramid pooling layers. The model achieved an area under the curve of 0.997, which is a sign of near-perfect classification accuracy. The inclusion of layers of spatial pyramid pooling enables the model to capture information at different scales, allowing it to classify cells with a wide range of abnormalities. The model achieves greater success in distinguishing cervical abnormalities at different stages because the convolutional neural network with the inception modules combines both low and high levels. Extremely specialized as this method is, it further enhances the accuracy of cervical cancer diagnosis, offering an effective way of detecting early cancer signs and ensuring that patients are treated in a timely and proper way.

Collectively, the results of these studies shed light on how the integration of advanced image processing methods, machine learning, and novel architectural approaches in facilitating cervical cancer diagnosis has the capability to make an impactful difference in the prognosis for early detection and treatment of the disease. Such innovations are most likely to increase classification accuracy, reduce human bias, and rationalize clinical procedures, thereby paving the way for substantial improvements in the early detection and treatment of cervical cancer. With the ongoing passage of time and clinical applications increasingly adopting such technologies in their operational paradigms, prospects for use across various healthcare setups, including the limited resource base, are set to increase. Further developments of more efficient and scalable models will be required to lessen the global disease burden of cervical cancer, hence enabling timely and accurate diagnosis among more women, which can contribute to better health outcomes and death reduction.

### 3.6 Innovative hybrid and ensemble approaches for cervical cancer detection

The advances in machine learning and deep learning have generated tremendous changes in cervical cancer diagnosis in terms of problems, such as accuracy, speed, and the potential for timely diagnoses. For example, Fekri-Ershad and Alsaffar (2023) propose a new model that integrates deep learning for feature extraction and a multilayer perceptron for classification. This model employs popular convolutional neural networks to scan cervical cell images. It is because of this that this model effectively combines the efficiency and feature extraction of deep learning with the precise classification of multilayer perceptron to prove the efficiency of the integration of different artificial intelligence concepts for diagnosis. The model with the impressive result shows the changing role of deep learning in screening cervical cancer through early diagnostic features by offering an automated and highly accurate diagnosis tool.

Similarly, Prusty et al. (2022) explore the application of the machine learning approach to diagnostic tests such as Hinselmann, Schiller, Cytology, and Biopsy. Among all the classifiers, the proposed random forest improved with stratified k-fold cross-validation and yielded the most accurate results for early cervical cancer prediction. This paper has shown that it is possible to apply machine learning algorithms to diagnose various types of cancer with high levels of precision, including in the early stages. By using stratified k-fold cross-validation, this method helps reduce overfitting and makes the model more applicable to different clinical situations. The successful use of random forest in this situation shows that it is very effective at handling complex relationships in medical data, which helps improve cervical cancer prediction with better classifiers. In addition to the existing hybrid and machine learning models, researchers have proposed new approaches to overcome the limitations of traditional diagnostic methods. For example, Kaushik et al. (2022) propose a deep learning approach for cancer risk assessment that aims to predict susceptible populations early. In this model, data is analyzed and visualized effectively to provide healthcare facilities with information concerning patients who stand at risk of getting cancer—information that may be useful in prevention and early detection. In addition to offering risk assessment, the system applies deep learning for predictive analytics to develop effective personalized care plans, enhancing patients’ quality of life by applying early interventions.

Apart from this, Ilyas and Ahmad (2021) contend with biases and overfitting issues that are common with single-classifier systems employing the ensemble method with the majority vote. The overall accuracy obtained with this method is 94% for the classifiers, i.e., decision tree, support vector machine, and logistic regression. Ensemble methods overcome the disadvantages of employing individual classifiers by merging the principal useful features, thus leading to greater reliability and stability. In medicine, ensemble learning methods, especially those that use majority voting, are popular because they make predictions more reliable and improve the system’s ability to apply to different situations, which is important for complicated diagnostic issues like diagnosing cervical cancer. In another study, Attallah (2023) introduced a new approach to overcome issues with the pre-segmentation step in computer-aided detection models. In this paper, to combine both handcrafted features with deep learning features, principal component analysis is utilized to reduce the complexity of the pre-segmentation steps in the classification process. The quadratic Support Vector Machine (SVM) achieved better performance compared to other models due to its ability to handle non-linear data, high speed, and strong generalization. By combining custom features with deep learning, the model can recognize both simple and complex patterns in cervical cell images, resulting in a stronger classification method. This innovation shows that the increase in the use of more fundamental image processing techniques in modern deep learning models may improve cervical cancer screening systems.

Combined, these works show how the use of hybrid, ensemble, and artificial intelligence approaches can overcome traditional challenges in cervical cancer screening. The use of mixed models, which incorporate multiple machine learning methods, deep learning, and other methods, along with lookup ensemble methods, not only helps in eradicating biases and overfitting but also yields higher diagnostic outcomes with greater efficiency Rojas et al. (2024). Additionally, this text shows how using deep learning can improve cancer risk assessment and optimize early steps in diagnosis, demonstrating that better cervical cancer detection systems can be created with artificial intelligence. Such advancement helps in early detection and, at the same time, gives the caregiver a chance for early intervention, which is critical. As artificial intelligence and machine learning work their way into clinical settings, these methodologies have the potential to dramatically change cervical cancer screening through improved accessibility, accuracy, and efficiency in different healthcare settings.

### 3.7 Advancements in AI and ML-based systems for cervical cancer screening: enhancing detection and risk prediction across diverse datasets

Cervical cancer remains one of the leading public health concerns in the world, and risk assessment and detection are critical for early intervention. Advanced technologies like machine learning (ML) and artificial intelligence (AI) have shown promising solutions to these issues, including early detection, enhanced screening accuracy, and improved clinical decision-making. For instance, Ratul et al. (2022) assess the efficacy of early risk prediction in cervical cancer cases using eleven different ML algorithms. Using hyperparameter optimization and grid search cross-validation, the algorithms achieved an accuracy of 93.33%. The present study illustrates the best application of conventional ML models for risk prediction by assessing their efficacy in classifying high-risk and low-risk individuals for early intervention.

Similarly, over the years, the available AI-based systems have also proved that cervical cancer screening is one of the areas that are set to benefit greatly from AI systems. Chen et al. (2021) propose CytoBrain, a system based on AI to allow correct cervical cell screening without human intervention. By using a CompactVGG network in CytoBrain, the tests showed that it could accurately and efficiently classify a large set of 198,952 cervical cell images. This system can be especially useful in mass screening since it allows processing a possibly large number of images in a short time and with high accuracy, thus relieving the cytologists and extending the opportunities for mass screening. AI’s use in such systems also eradicates human bias, thus bringing efficiency to diagnosis procedures and eradicating human error. Yang et al. (2024) reported more recent studies in China on AI-improved cervical cancer detection. This study looked at an AI-assisted system for examining cells, comparing it to the standard method called liquid-based cytology (LBC) along with HPV testing. The results showed that AI accuracy was even higher than the accuracy of LBC alone with HPV co-testing in the detection of HSILs and LSILs. This suggests that specific AI systems could potentially enhance the effectiveness of colposcopy and increase its device accuracy while decreasing the need for conventional but formal time-consuming screening. This predictability of AI’s ability to pick out even minor precancerous changes to cervical cells places it at the forefront of cervical cancer prevention.

However, some of these issues, such as scalability and opportunities for application to deprived regions, remain unresolved. Soni and Soni (2021) meet these challenges by examining online and offline ML, particularly the CNN-CRF model. Particularly in developing countries like India, this model has effectively identified cervical cancer from medical images. This work also demonstrates that we can implement AI models, particularly CNN-based frameworks, in settings with limited trained human personnel and diagnostic instruments. Flexibility in AI systems is essential for cervical cancer screening, as it can be implemented across a diverse population, increasing the worldwide utilization of cervical cancer screening and reducing the global threat of cervical cancer. Furthermore, Ahmed et al. (2024) examine the problem of generalizability in AI models for clinical practice. It was also using a multi-stage AI pipeline on a heterogeneous set with a total of 17,013 images. The results indicated that the issues of device heterogeneity were decisive for lower AI models’ performance rather than regional characteristics. This insight is valuable because it emphasizes the need to consider ways to make AI systems resilient enough to function optimally, regardless of the type of medical device or imaging system that they interface with. It also showed that retraining models in incremental steps can be used to manage performance problems due to infrequent changes in medical devices. Adjusting artificial intelligence models to fit different clinical settings is crucial for their effectiveness and helps maintain their ability to deliver accurate results in those settings.

The emergence of newer and adaptive technologies in ML and artificial intelligence will indeed revolutionize cervical cancer screening by improving its precision, duration, and the reach of countless patients owing to technology. These technologies reduce the necessity of manual labor, relax the workload on the medical personnel, and expand cervical cancer screening’s accessibility across different communities. This intrinsic property of AI systems to learn from particular contexts makes the technology an excellent one, as it offers the possibility for optimizing early detection and the delineation of clinical care as well as boosting the ability to achieve better patient outcomes for a wide variety of clinical settings. However, AI can be successfully applied to actual clinical practice only by overcoming device compatibility issues, data quality, and ethical issues. That is why further research and fine-tuning are required to make AI approaches universal and effective in cervical cancer prevention.

Some of these technologies are tackling important problems in areas with limited resources, dealing with different types of data, and predicting risks early, which helps provide effective and trustworthy solutions for finding and managing cervical cancer.

### 3.8 AI-driven innovations in cervical cancer detection: a comprehensive review of DL and ensemble methods

There is a need to offer better and more accurate models for cervical cancer screening for LMIC healthcare facilities and for those countries that have a highly developed healthcare system. There is a major issue regarding the early stages of cervical cancer—the detection of precancerous lesions—and ML technology has a solution for this. One such achievement is the Deep Metric Learning (DML)-based approach offered by Pal et al. (2021). This system eliminates the need to mark the cervix boundary by using the powerful feature extraction capabilities of strong convolutional networks. These descriptors are utilized to train a KNN classifier, which has the possibility of detecting cancerous as well as non-cancerous tissue with an extremely high degree of effectiveness. This model is especially helpful as it increases specificity in disease detection and does not sacrifice sensitivity, making it a valuable tool for early cervical precancer detection where other procedures like manual examination and cervix boundary marking may not be possible. Furthermore, the paper offered by Manna et al. (2021) employed and enhanced cervical cancer screening by using an ensemble learning technique to fuse three of the most widely used convolutional neural networks, Inception v3, Xception, and DenseNet-169. A fuzzy rank-based fusion technique follows this ensemble model, which sums up the probabilities provided by different classifiers. This ensemble model seems to be superior to most of the models offered in the literature, and the results show good potential for improving cervical cancer detection by fusing a number of classifiers. The use of fusion techniques also allows for error minimization and improvements in the reliability of predicting which features are important for clinical decision-making.

On the class imbalance problem owing to the heterogeneous number of data points in medical datasets, Tanimu et al. (2022) propose a new way to combat this problem to enhance the efficacy of the model. This model uses a powerful Decision Tree (DT) classifier that includes techniques to effectively address the class imbalance issue and to choose the most important features for classification. This model is particularly suitable for situations where the proportion of cancerous cases is significantly smaller than non-cancerous cases, which is common in cancer diagnosis and research. Additionally, Song et al. (2022) prove the applicability of DL for cervical and endometrial cancer classification using whole-slide images (WSIs) of tissue samples. The study finds that DL classifiers are effective if WSIs are broken down into small images such that classification is easily enabled. The results show that DL can improve diagnosis, especially for cervical cancer, because it needs detailed large medical images to tell different types of cervical cancer apart and to identify where adenocarcinomas come from. Fang et al. (2022) proposed DeepCELL, a deep convolutional neural network, for the classification of cervical cytology images. The article described that the multiple kernel approach uses various kernels of different sizes to extract features from an image, which helps the system better identify cervical cells. This study has utilized proven cervical cytology data such as Herlev and SIPaKMeD; DeepCELL’s accuracy reached 95.63%—a performance superior to other approaches highlighted herein. The above results indicate that DL can be utilized to reduce the margin of error incorporated during screening cervical cancer, enhance the diagnostic reliability, and reduce the reliance on skilled cytologists. Besides, this model provides cervical cancer diagnosis with a more automated, efficient, and reliable process that will greatly assist detection and early detection.

Altogether, the presented investigations show the significant progress achieved in cervical cancer recognition by using AI and ML. The use of deep metric learning frameworks and ensemble methods, along with techniques for handling data imbalance and improving accuracy, shows great promise for better early detection and diagnosis of cervical cancer. The adaptation of these technologies in clinical practice can decrease the number of diagnostic errors and improve operational efficiency and patient care. However, as these learning models develop, problems with the quality of data, understanding how the models work, and fitting these models into existing clinical practices will create major challenges in detecting and preventing cervical cancer worldwide.

AI-driven methods have enhanced cervical cancer detection, as well as classification, significantly. Techniques such as DL, ensemble models, and feature optimization can enhance diagnostic accuracy, handle data imbalances, and improve specificity and sensitivity. These innovations are scalable and provide solutions that are efficient enough for early detection, allowing for improved clinical outcomes.

## 4 Smote TOMEK for handling class imbalances

The cervical cancer dataset suffers from severe class imbalances, which contain around 96% of non-cancerous data and only 4% of cancerous. This serious issue can lead the model trained with this data to classify inappropriately, resulting in poor detection. SMOTE (Synthetic Minority Over-sampling Technique) addresses this issue by integrating both oversampling and under sampling techniques. The SMOTE technique helps in classification by creating synthetic samples for the minority class data by interpolation between an instance and its nearest neighbors. We discover and eliminate Tomek links—pairs of examples from opposing classes that are in close proximity—after adding the synthetic samples. This stage aids in eliminating noisy or marginal samples and minimizes class overlap. This results in enhanced training data, which enhances the classifier’s potential to identify more accurate boundaries among the two categories Tanimu et al. (2022). The proposed model follows the steps shown in Algorithm.

Algorithm Synthetic Minority Oversampling Technique (SMOTE).**Input:***X*_min: Collection of minority class instances.*N*: the total number of synthetic samples to be created
k: Number of closest neighbours (often 5)**Output:***X*_augmented: Inclusion of synthetic instances to minority class samples**Procedure:**1. For each sample *xi* in *X*_min:
        1.1 Identify the *k* nearest neighbours of xi within *X*_min utilising Euclidean distance. Select *n* neighbors at random from the *k* neighbours according to the specified oversampling ratio.
2. For each chosen neighbour *xj*:
       2.1 Create a synthetic sample *x*_syn as outlined below: *x*_syn = *xi* + random(0,1) * (*xj* − *xi*), where random(0, 1) denotes a random number within the interval [0, 1].
3. Repeat steps 1–2 until a cumulative total of *N* synthetic samples is generated.
4. Integrate all generated *x*_syn into the original minority class set to create the final augmented dataset *X*_augmented.

RQ1. How do AI- and ML-based diagnostic tools perform compare to traditional methods in terms of accuracy, sensitivity, and specificity for detecting cervical cancer?

AI and ML-based tools, for instance, have proven to achieve much more sophisticated detection of cervical cancer than a traditional diagnostic process like Pap smears and HPV testing. EfficientNetB0, Cervical Net, and ResNet-50 use deep learning and advanced computing to analyze medical images and patient records. Unlike traditional approaches, which depend on trained cytologists and are prone to human errors due to the manual evaluation of samples, AI models automate the diagnostic process with minimal dependence on human expertise. This is an additional benefit of AI-based approaches, particularly in low-resource settings where specific personnel and diagnostic tools are limited. It can analyze large amounts of data at a rapid rate, ensuring prompt detection and diagnosis of cervical abnormalities. These instruments are also scalable. They can, therefore, be implemented in so many healthcare environments with so many different resource availabilities. Furthermore, they give very high sensitivity for the identification of small cervical cancer and cancer precursors. As methods capable of filling the shortcomings of traditional approaches, AI and ML are on the verge of transforming cervical cancer screening and diagnosis to be more widely available, precise, and effective on a global scale.

RQ2. What are the primary barriers to the adoption and successful integration of AI-based diagnostic tools in cervical cancer screening programs in LMICs?

Numerous challenges have hindered the extensive use of AI-based tools in LMICs. One of the major challenges here is the absence of properly labeled datasets needed for training most AI systems. Annotated medical data is a process that requires specific skills, is time-consuming, and requires significant resources. Many of these settings are not resource-efficient, and therefore annotating their data becomes impractical. It reveals that AI deployment is equally challenging due to a lack of sufficient healthcare facilities, such as diagnostic tools and poor internet connection. Lack of clinicians and resistance to change among healthcare providers, coupled with inadequate training in AI, are other challenges. While concerning for patients’ privacy, standards such as GDPR (General Data Protection Regulation) and HIPAA (Health Insurance Portability and Accountability Act) result in data sharing limitations that are difficult to overcome when gathering various datasets needed for model development. LMICs worsen privacy issues due to a lack of awareness about secure data management. Such barriers cannot be surmounted without unique solutions, including federated learning, which can make the models themselves portable and trainable while still preserving the data. Another intervention is educating human resources in clinical practice and investing in public health education to mainstream AI in LMICs.

RQ3. How can AI and ML techniques address the challenges of class imbalance, data privacy concerns, and model interpretability in cervical cancer detection, and what impact do these solutions have on model performance in real-world clinical settings?

The application of AI and ML can help solve three primary issues related to the utility of sensitive diagnostic tools in cervical cancer screening, namely class imbalance and privacy. Class imbalance is a situation where a data set contains few malignant cases while the models are inclined to relapse on the dominant classes instead of the minority ones. Adaptive SMOTE Tomek is a strong technique to handle this problem by combining synthetic data generation for underrepresented classes with noise reduction for mislabelled or ambiguous samples. This ensures that the model trains in a fairer manner, thereby increasing sensitivity and accuracy in detecting malignant cases. Privacy concerns can be addressed by federated learning, which is a decentralized model training framework that eliminates the need for data centralization. Instead, models are trained locally on individual datasets with only updates on the model shared across sites. This will work in a way to respect regulations like GDPR and exploit more diverse datasets towards more generalized models. Together with federated learning, Adaptive SMOTE Tomek will improve AI model fairness and performance while building trust among stakeholders for integrating AI in clinical workflows across the globe in a secure manner.

## 5 Findings and discussion

By thoroughly checking each paper that discussed using AI for cervix cancer detection (a total of 63), Arguably there is a marked improvement in the technical side of AI applications for cervical cancer screening. Still, the step from high accuracy algorithms to the day-to-day implementation in the clinic must be scrutinised for what comes next four areas: dataset properties, validation methods, completeness of evaluation metrics and constraints at the time of deployment in an actual clinic. Adaptive SMOTE Tomek and federated learning propose strategies to overcome these challenges, training robust models while maintaining data privacy and standardization. Hybrid approaches, combining handcrafted and deep features, have improved interpretability and reliability, which are vital for clinical acceptability Gururaj et al. (2025).

### 5.1 Benchmarks for testing and medical photography modalities

The diagnostic capability and versatility of artificial intelligence (AI) tools in healthcare are highly contingent on the structure and integrity of the datasets from which they are derived. Most studies reported here use public, standardized benchmark repositories with private clinical cohorts: Standard, Publicly Available Datasets: Majorly, cell-level identification algorithms have been trained with publicly available reference data like Herlev, which includes $2,010$images, SIPaKMeD with 4,049images, and even CRIC with 11,551images. Even though, these sets provide standardized answers, their limited sample sizes, and single-pre-segmented cell compositions do not represent well, the real-life conditions such as noise and multiple-cell backgrounds that are commonly seen in routine liquid-based cytology (LBC) or conventional Pap smear preparations. Clinical Whole-Slide Images (WSI) and Colposcopy: Those works that involve the use of whole Slide image scanning (WSI) or cervicography/colposcopy data may have more practical usefulness but they are plagued by class imbalances at a serious level. Only very small proportions of the patients with cervical intraepithelial neoplasia (CIN2+) are detected in cervical cancer screening programs thereby introducing extremely uneven class proportions which negatively affect performance of classifiers that are not corrected for the class distributions.

### 5.2 Critical appraisal of validation methods and data leakage

This methodological review identified some levels of validation malignancy in the synthesized literature: Validation protocols: More than 65% of the primary research in the review only employed k-Fold or Stratified K-Fold validation modalities on single Source datasets. Although indispensable for model hyperparameter optimization, single-cohort validation will often report overoptimistic performance measures owing to known background features in a particular scanner, staining protocol, clinic population and so on. Pre-processing data leakage: Quality appraisal Domain 3 (Validation Appraisal) illuminated the frequency of data leakage during pre-processing: a minority of studies used synthetic oversampling protocols (e.g., SMOTE) or image transformations before dataset partitioning into train-test files, allowing the validation fold to absorb synthetic distortions created from the test set distribution. This compromises validation results, vastly inflating accuracy measures. External and Multi-center validation: only a minority of studies employed independent external validation on other hospital cohorts or multi-scanner test beds. Absent external cross-site validation, claims that AI exceeds experienced cytotechnologists, or traditional work flows in large biopsy closed populations, are unsubstantiated.

### 5.3 Completeness of evaluation metrics

A fatal flaw detected during the review: a general dependence on Global Accuracy measures for performance summaries: the accuracy paradox in healthcare data: in imbalanced cervical screening databases, where CIN+ diagnoses occur in less than 5% of samples, a formal model guessing all negatives still achieves over 95% accuracy, but fails to identify detecting CIN+ lesions altogether. Comprehensive metric requirements: There can be no rigorous diagnostic evaluation without cross-equal reporting of Sensitivity (Recall) Specificity Positive Predictive Value, False Negative Rate and area under the ROC curve. Screening models must achieve high sensitivity in the absence the many false negatives would dominate a final cost–benefit calculation, while secondary triaging guides require high specificity.

### 5.4 Methodological limitations and real-world implementation

Promised computational processes becoming part of working systems bring along several challenges:

#### 5.4.1 High computing requirements versus edge deployment

Models with Vision Transformers (ViTs) or deep CNNs have a large number of parameters and because of this a very high need for GPU computing power which makes it impossible to deploy such systems in low-resourced small clinics—the very places where a point-of-care digital screening would be most useful.

#### 5.4.2 Privacy-preserving methods (federated learning)

Decentralized approaches like federated learning (FL) that allow multiple organizations to train a single medical AI while keeping their data private are a promising way to address issues like data scarcity and privacy. Yet, practical deployment of FL is hindered by communication costs, clinical data that is non-independently and identically distributed (non-IID) across sites, and vulnerabilities to membership inference attacks.

##### 5.4.2.1 Class imbalance solutions (Adaptive SMOTE-Tomek)

Techniques which combine noise removal and data augmentation in minority class such as Adaptive SMOTE-Tomek were found to A lot reduce the dominance of the majority class. Though, one has to be very careful while using such techniques not to interfere with the diagnosis.

#### 5.4.3 Methodological evaluation summary table

Table 3 shows a comparative overview of methodological elements across cervical cancer diagnostic literature.

**Table 3:** Comparative overview of methodological elements across cervical cancer diagnostic literature.

Dimension	Standardized benchmark literature	Advanced multi-center/WSI literature	Proposed/theoretical frameworks
Primary datasets	Herlev, SIPaKMeD, CRIC (single-cell/cropped)	Multi-hospital WSIs, colposcopy cohorts	Synthetic/distributed multi-site data
Validation strategy	k -fold cross-validation (single dataset)	Multi-center/multi-scanner external sets	Simulated local validation sets
Target metrics	Accuracy, precision, F1-score	Sensitivity, specificity, AUC-ROC, FNR	Convergence rate, differential privacy
Key constraints	Class imbalance, synthetic image overfitting	Staining variations, annotation bottlenecks	Communication overhead, non-IID data
Clinical readiness	Low (limited to pre-segmented cells)	Moderate (requires clinician verification)	Conceptual/experimental phase

### 5.5 Understating clinical impact

In absence of multi-center, prospective validation of AI approaches for diagnosis of cancer on one hand and their performance compared to traditional methods under real-life conditions Then again, we have to be very careful not to overemphasize clinical claims of AI methods, e.g., their ability to detect cancer in images better than humans leading automatically to longer survival. What we can and have to say now is that AI methods should best be considered as tools which help clinicians by doing part of the screening work, reduce fatigue errors, etc. They will definitely not completely replace screening programs led by medical doctors anytime soon.

## 6 Conclusion and future works

The study highlights the significance of advanced cervical cancer screening technologies, particularly in LMICs. Exceptional diagnostic performance has been displayed by AI models such as EfficientNetB0 and Cervical Net; their performance has made it possible to significantly reduce dependency on specialized staff and increase efficiency in cervical cancer screening. Hence, these findings open up an avenue for early detection that ultimately allows for early intervention and, consequently, better patient results. Despite these successes, there are challenges that counterbalance the consideration of AI-driven solutions. Some of these include data privacy issues, class imbalances, and the limited interpretability of complex AI models. These are barriers to the adoption of AI-driven solutions. Innovative approaches to deal with these challenges include federated learning and Adaptive SMOTE Tomek. Federated learning is privacy-compliant by decentralizing the learning process. Adaptive SMOTE Tomek balances class ratios and significantly improves the model’s performance in the underrepresented category. The testing of these frameworks in various clinical settings will serve as the backbone for future research with the aim of validating their generalizability. Explainable mechanisms of AI can be integrated so that healthcare professionals can understand precisely how the decision-making process took place. Second, the inclusion of more extensive annotated datasets for diverse demographic features and pathological descriptions will improve model utility and precision. These moves will position AI as a necessity for cervical cancer prevention and care for the world at large.
